# Could β-glucans enhance the immune response to SARS-CoV-2 vaccine by inducing trained immunity and boosting neutralizing antibody production?

**DOI:** 10.3389/fimmu.2026.1675094

**Published:** 2026-02-27

**Authors:** Márjorie de Assis Golim, Patrícia Valério Orlandi Falbo, Aline Márcia Marques Braz, Rafael Plana Simões, Rejane Maria Tommasini Grotto, Beatriz Furtado Pegatin, Jaime Olbrich-Neto, Rui Seabra Ferreira

**Affiliations:** 1 Graduate Program in Research and Development, Medical Biotechnology, Botucatu Medical School, São Paulo State University (UNESP), Botucatu, São Paulo, Brazil; 2 Flow Cytometry Laboratory, Applied Biotechnology Laboratory, Clinical Hospital of Botucatu Medical School, São Paulo State University (UNESP), Botucatu, São Paulo, Brazil; 3 Graduate Program in Clinical Research, Botucatu Medical School, São Paulo State University (UNESP), Botucatu, São Paulo, Brazil; 4 Department of Bioprocess and Biotechnology, School of Agriculture, Sao Paulo State University (UNESP), Botucatu, São Paulo, Brazil; 5 Plant Protection Department, School of Agriculture, Sao Paulo State University (UNESP), Botucatu, São Paulo, Brazil; 6 Pediatrics Department, Botucatu Medical School, São Paulo State University (UNESP), Botucatu, São Paulo, Brazil; 7 Center for the Study of Venoms and Venomous Animals (CEVAP), São Paulo State University (UNESP), Botucatu, São Paulo, Brazil

**Keywords:** beta-glucans, Covid-19, SARS-CoV-2, trained immunity, vaccines

## Abstract

β-Glucans stimulate the immune system, training it to recognize and respond to antigens, which bolsters immunity, including to vaccines. The present study evaluated the capacity of β-glucans to stimulate immunity subsequent to primary vaccination for SARS-CoV-2 with ChAdOx1. Thirty-four SARS-CoV-2-non-immune men (18–49 years) were split into two groups: one receiving 500 mg of oral insoluble yeast β-glucans daily (the glucan group) and the other a placebo (the control group). The supplementation period lasted fourteen days in total, including seven days before and seven days after the initial vaccine dosage. A series of blood samples were collected at three time points: M1 (prevaccination); M2 (30 days after the first vaccination); and M3 (30 days after booster). A lateral flow immunoassay was used to qualitatively identify IgM and IgG against the virus. The levels of antigen-specific IgG anti-spike (S1), receptor-binding domain (RBD), and nucleocapsid (N) were quantified using a LEGENDplex assay. The NeutraLISA assay was used to evaluate the neutralizing antibodies (NAbs). Statistically significant results were defined as those with *p* < 0.05. Both groups produced similar amounts of NAbs after the first vaccination (M2). However, the glucan group had higher levels in M3, with a more uniform distribution. Furthermore, the levels of anti-S1 IgG in M2 exhibited elevated concentrations, indicating a significant positive correlation with NAbs levels obtained post-second dose (M3). In contrast, individuals who had immunity to common cold human coronaviruses (HCoVs), evidenced by the presence of IgG anti-N in M1 were associated with IgG anti-S1 only in M3, not correlated with NAbs levels. This finding indicates that cross-immunity from other HCoVs did not accelerate or direct the humoral immune response as was observed in the glucan group. Therefore, it can be inferred that the β-glucan supplementation was more effective than immunity from other HCoVs. The capacity of β-glucan to induce trained immunity (TRIM) has the potential to augment immune responses, thereby modifying antibody production in response to the vaccine stimulus. Future studies should evaluate the potential of β-glucan as an adjuvant to vaccines, especially in children, the elderly, and immunocompromised individuals. They should also assess long-term immunity and cross-protection.

## 1 Introduction

Immunization represents a cornerstone strategy in the fight against pathogenic threats. The emergence of the novel coronavirus significantly accelerated the development of vaccines and antivirals, marking an unprecedented milestone in biomedical research. In parallel, numerous research institutions sought to identify effective immunizing agents by employing a wide range of technological approaches. At the onset of the COVID-19 epidemic in China, in the absence of specific antivirals or effective vaccines, health authorities turned to Traditional Chinese Medicine (TCM) as a complementary approach to clinical management. Classical herbal formulas such as *Lianhua Qingwen*, *Qingfei Paidu Tang*, and *Shufeng Jiedu* were incorporated into national guidelines for mild and moderate cases, with preliminary studies suggesting symptomatic improvement and a potential reduction in disease progression (1, 2). Concurrently, laboratory investigations identified natural compounds—including polysaccharides, flavonoids, and alkaloids—with antiviral and immunomodulatory properties. As vaccines advanced in late 2020, the role of TCM shifted toward supportive care, immune modulation, and the management of post-infection sequelae. Recent systematic reviews and meta-analyses have consolidated evidence that TCM may serve as an adjunctive therapy in different stages of COVID-19, although robust clinical trials remain necessary to confirm its efficacy as a vaccine adjuvant (3–6).

The ChAdOx1 nCoV-19 vaccine, developed using a chimpanzee adenovirus vector, expresses the SARS-CoV-2 spike (S) protein on its surface and has demonstrated the ability to elicit strong cellular and humoral immune responses. Cellular immunity is typically induced by day 14 following the first dose, while humoral immunity peaks with the production of specific antibodies around day 28. Moreover, neutralizing antibody titers are consistently observed after administration of the second dose, confirming the vaccine’s capacity to generate durable protective immunity against SARS-CoV-2 (7–10).

The predominant objective of the majority of immunization programs is the induction of adaptive immunity. However, there is an ongoing investigation into approaches that target the strengthening of the innate immune system. The strategic intent of these approaches is to enhance the immune response via distinct pathways (11–16). The innate immune system is the first line of defense against infection, essential for the early recognition of invading pathogens and the subsequent activation of a pro-inflammatory response (8). Although immune memory is a peculiar feature of the acquired immune system, activation of the innate system can also result in a greater capacity to respond to subsequent stimuli. First proposed by Netea, Quintin, and Van der Meer in 2011, “trained immunity” (TRIM) describes how innate immune cells can develop a memory-like response (17). This phenomenon is based on the premise that a prior stimulus, such as a vaccination or infection, induces lasting phenotypic changes in these cells, allowing them to mount a more efficient response to secondary or heterologous stimuli (17–23).

The Bacillus Calmette-Guérin (BCG) vaccine and β-glucans are among the main and most studied TRIM inducers (23–26). β-glucans are a heterogeneous group of polysaccharides that are abundant in the cell walls of yeasts, bacteria, algae, and fungi. Those of fungal origin, such as those extracted from *Saccharomyces cerevisiae*, are considered efficient immunomodulators due to their more vigorous effects on the immune system (27–30). These natural biomolecules consist of a central linear core of glucose units linked at the β- (1, 3) position and side chains of varying sizes made of glucose linked at the β- (1, 6) position (29). Innate immune cells undergo a rewiring of the epigenome and metabolism by β-glucan, resulting in the induction of TRIM. Consequently, these cells exhibit a heightened capacity to elicit stronger, faster, and/or qualitatively distinct transcriptional responses when exposed to subsequent stimuli (18–31).

Recent studies suggest that β-glucan-induced TRIM increases the efficacy of a number of vaccines, potentially strengthening antibody responses. This phenomenon has implications for immune health and disease resistance (23, 32, 33). This approach may constitute a valuable strategy in vaccination protocols against diseases such as SARS-CoV-2 (19, 31, 34–37). It has been demonstrated that certain fibers, including but not limited to mekabu fucoidan and inulin-type fructans, have the capacity to enhance immune responses to vaccines for various diseases, such as influenza and hepatitis B. These findings are consistent with the properties of other natural compounds that have been shown to possess beneficial health effects. These strategies may help control epidemics (38–41).

In light of the well-established safety of oral administration of β-glucans as a nutritional supplement and their recognized benefits in regulating the immune system, the conduction of clinical studies is encouraged (26, 43). β-glucans are stable and simple to take orally, and their safety has been confirmed by the FDA and the European Food Safety Authority (36, 44–46). Furthermore, the cost of obtaining them is relatively low, and the risk of adverse effects is minimal (47). They are approved as adjuvant drugs in several countries and due to their immunomodulatory effects. Studies have reported their effectiveness as adjuvants in vaccines against various diseases (36, 40, 41, 44, 46).

A preliminary study conducted by our group (unpublished) investigated the ability of β-glucans to induce TRIM in individuals indicated to receive the hepatitis B booster vaccine, confirmed by the absence of protective serological levels. No significant differences emerged between placebo and glucan groups, leading us to hypothesize that pre-existing immunological memory accounted for the rapid clonal expansion and antibody production in both. Consequently, the present study focused on primovaccination in SARS-CoV-2–naïve subjects to examine whether β-glucans could enhance immune responses in the absence of prior memory.

## 2 Methods

### 2.1 Volunteers and sample collection

**Figure 1:**
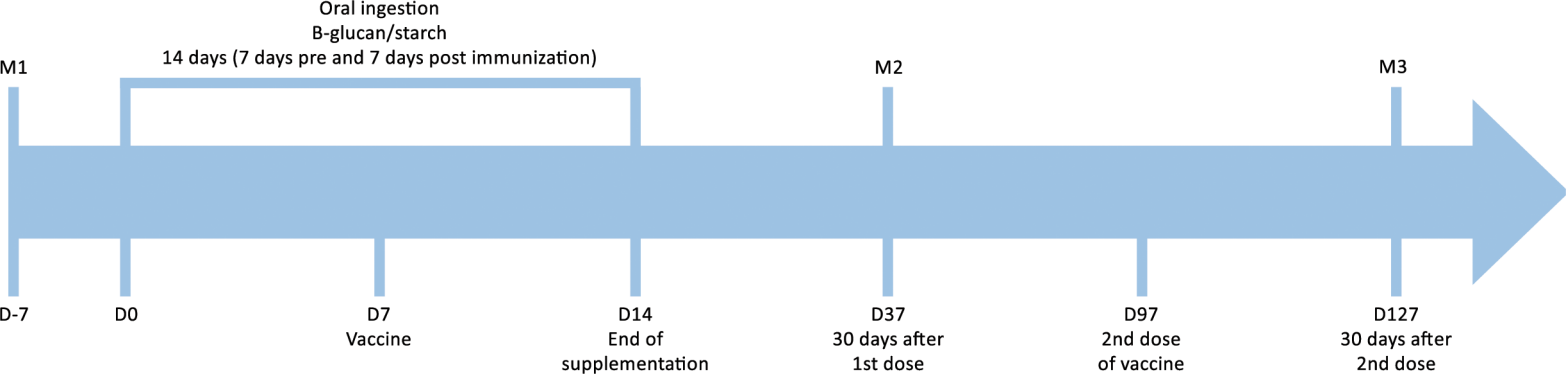
Study design: supplementation, vaccine, collection of samples for laboratory evaluations. M1 (Moment 1): sample collection 7 days before supplementation for serological screening; M2 (Moment 2): sample collection 30 days after the first dose of vaccine; M3 (Moment 3) sample collection 30 days after the second dose of the vaccine; D: day.

A randomized, blind, placebo-controlled pilot trial was conducted on volunteers who received β-1,3-1,6-glucan derived from *Saccharomyces cerevisiae* yeast or a placebo (starch) seven days before and seven days after receiving the ChAdOx1 nCoV-19 vaccine (**Figure 1**). The utilized β-glucan is characterized by its insoluble nature, with a purity level exceeding ≥70.0%, and an average particle size ranging from 70 to 100 μm. The compositional specifications consist primarily of carbohydrates, principally in the form of β-glucans, accompanied by minimal quantities of protein, fat, and ash.

The study population comprised male subjects between the ages of ≥18 and <50 years of age who volunteered to donate blood and did not have a history of previous infection with the SARS-CoV-2 virus. Furthermore, the subjects did not manifest any concomitant comorbidities, including autoimmune, infectious, hematological, or oncological diseases. Given the established influence of hormonal, genetic, and environmental factors related to sex on immune responses (48, 49), the participants were limited to men to promote sample homogeneity and minimize potential biases. The local research ethics committee approved the study. Participants were excluded from if they indicated an intention to discontinue their participation, did not attend a sample collection, reported improper adherence to the supplementation regimen, or exhibited specific anti-SARS-CoV-2 antibodies in the prevaccination sample (M1).

The initial stage entailed serologic screening to distinguish non-immune individuals, herein referred to as naïve. In the serological screening conducted on forty-six participants (n=46), twelve (n=12) were identified with positive serology for SARS-CoV-2, indicating previous infection, possibly asymptomatic, as the participants were unaware of the infection. Consequently, the involvement of these 12 subjects was terminated, and the study continued exclusively with non-immune individuals.

The thirty-four participants were divided into two groups through simple randomization (42) using a random number table, with even numbers allocated to the Control Group and odd numbers allocated to the Glucan Group. The Control Group received starch capsules (placebo) at a dosage of 500 mg/day (n = 17). The Glucan Group was administered β-glucan supplement capsules at a dosage of 500 mg/day (n = 17). Both groups received their respective treatments for a period of two weeks, with the first week serving as the baseline and the subsequent week serving as the post-immunization follow-up. The study’s design is illustrated in **Figure 1** above. The researchers then monitored the participants for symptoms associated with the vaccination, as well as for adverse effects related to the supplementation. The safety of the oral intake of 500 mg was previously tested. A dose of up to 1275 mg/day is considered safe for adults and children over 12, according to the recommended European limit (45). A clinical trial by Leentjens et al. demonstrated this by administering 1000 mg/day of β-glucan (in two 500 mg doses) over seven days to assess its impact on the human innate immune response (44).

A series of peripheral blood samples were collected at three time points (moments=M). Prior to the administration of the vaccine (M1), the objective was to screen and identify individuals who were not immune to SARS-CoV-2 (naïve). The baseline, or day zero (D0), was defined as the initiation of supplementation with β-glucan or placebo. Subsequent to a week of supplementation, the initial dosage of the ChAdOx1 vaccine (AstraZeneca/Oxford) was administered. The vaccine is currently available within the Brazilian Unified Health System (SUS) and has received approval for utilization from the National Health Surveillance Agency (ANVISA). The administration of the vaccine occurred as part of an effectiveness study of the ChAdOx1 vaccine (AZD1222) that was carried out in the city of Botucatu, São Paulo, Brazil, in May 2021. Therefore, all participants received their vaccine doses on the same dates (16). Oral supplementation was maintained for a duration of seven days following vaccination (D14). The second collection was performed 30 days after the first dose of the vaccine (M2 = D37). The second dose of the vaccine was administered 90 days after the initial dose (D97) in August 2021. The third collection was performed 30 days after the second dose of the vaccine (M3 = D127). At predetermined intervals, 5-milliliter samples of peripheral blood were collected in EDTA tubes.

### 2.2 Anti-SARS-CoV-2 antibody profile pre- and post-vaccination

The anti-SARS-CoV-2 antibody profile was conducted for two primary objectives: first, to identify non-immune (naïve) volunteers, and second, to assess the levels of antibody response induced by the primary vaccine. Plasma screening was performed using two methods: (1) An immunochromatography test was used to detect anti-SARS-CoV-2 IgM/IgG (ECO Diagnosis^®^). Venous blood collected in EDTA is added to the test device, followed by specific diluent buffer, and the reading is performed within 15 minutes. If antibodies are present, they interact with a conjugated gold particle complex and migrate in the membrane. In the presence of antibodies, a colored test line is visible. The control line confirms the test’s functionality. (2) The concentration of IgG antibodies specific to the SARS-CoV-2 Spike (S1), receptor-binding domain (RBD) and nucleocapsid (N) was determined using the LEGENDplex^®^ SARS-CoV-2 Serological IgG Panel (Biolegend™), an immunoassay based on beads (multiplex) that can differentiate between immunoglobulins directed against different viral antigens. The detection process was executed using the FACSCalibur^®^ instrument (BD™), and the quantitative outcomes were expressed in ng/mL. The assays were performed in accordance with the manufacturer’s instructions.

### 2.3 Determination of neutralizing anti-SARS-CoV-2 antibodies

Neutralizing antibodies (NAbs) were determined with the Enzyme-Linked Immunosorbent Assay NeutraLISA SARS-CoV-2 kit (Euroimmun™) according to the manufacturer’s instructions. Plasma samples were diluted and incubated with the S1/RBD domain of the Spike protein from SARS-CoV-2. The NAbs present in the sample compete with the ACE2 receptor for the binding sites of the S1/RBD proteins. The biotinylated ACE2 bound to S1/RBD was detected by incubating the wells with peroxidase-labelled streptavidin for 30 min at room temperature. The peroxidase reaction was subsequently subjected to visualization through the use of a 3,3’-diaminobenzidine (TMB) solution for a period of 15 minutes at ambient temperature. Subsequent, a stop solution was incorporated and the optical density (OD) was measured at 450 nm. A percentage of inhibition, herein denoted as “%IH,” was determined through the following calculation: %IH = 100% - (extinction test sample/100% - blank mean).

### 2.4 Immunophenotypic characterization of B lymphocyte subpopulations

The frequencies of B lymphocyte subpopulations were evaluated at three collection times using the following markers: CD45-PerCP (clone 2D1, BD Biosciences), CD19-FITC (clone HIB19, BD Biosciences), CD27-PE (clone M-T271, BioLegend), and CD38-APC (clone HB-7, BioLegend). In order to ensure the accuracy of the findings, unstained and isotype controls were performed for each of the fluorescence channels utilized in the analyses. Analyses were performed using a FACSCalibur^®^ flow cytometer (BD) with four fluorescence detectors. Data acquisition and analysis were conducted using CellQuest™ and FlowJo™ software with diverse analytical strategies depending on the subpopulation of interest: CD45+/CD19+: total B lymphocytes, CD45+/CD19+/CD27+/CD38-: memory B cells, and CD45+/CD19+/CD27+/CD38+: plasmablasts.

### 2.5 Statistical analysis

Statistical analysis was performed using R software^®^ version 4.4.2 and GraphPad Prism^®^ version 6.01. Initially, the data were subjected to the Shapiro-Wilk normality test. Variables with a symmetrical distribution were expressed as mean and standard deviation as measures of central tendency and analyzed using the student’s t-test and paired t-test. Variables with an asymmetrical distribution were expressed as median and semi-interquartile range and analyzed using the Mann-Whitney and Wilcoxon (paired) tests. Pearson and Spearman correlation tests were also performed, and *p* values less than 0.05 were considered statistically significant.

## 3 Results

**Figure 2:**
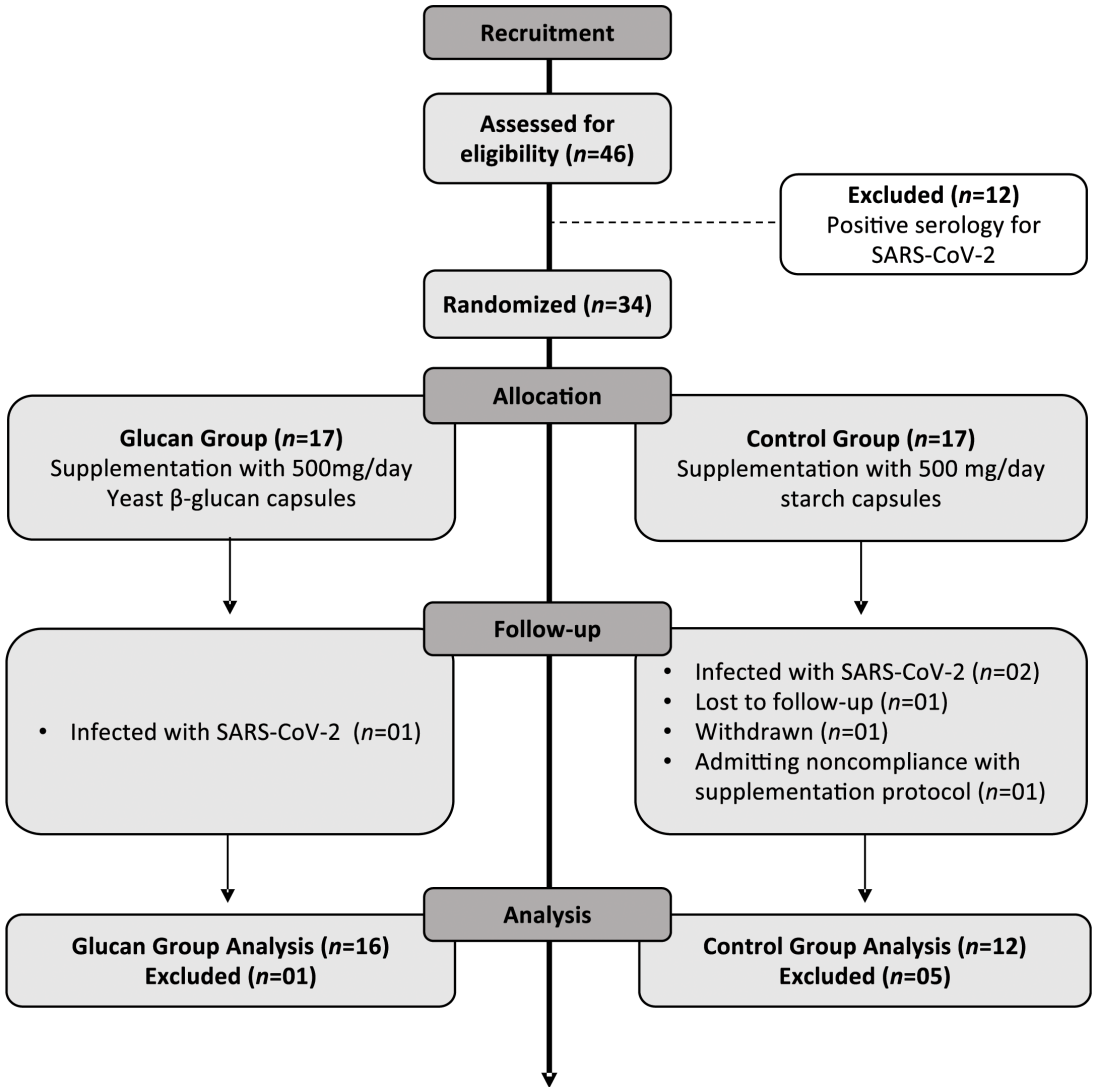
CONSORT diagram of participant selection and follow-up.

Serologic screening of forty-six participants identified twelve (n=12) with prior SARS-CoV-2 exposure, who were excluded. The remaining thirty-four naïve male subjects were randomly allocated to the supplementation groups as described (Glucan Group n=17; Control Group n=17). It is noteworthy that there were no adverse reactions reported by the participants during the supplementation period. Prior to the administration of the second vaccine dose, three participants were infected with SARS-CoV-2 and were therefore excluded from the group analysis (n=1 Glucan Group; n=2 Control Group). During the supplementation period, three participants from the control group were excluded from the study: one for withdrawing, one for admitting noncompliance with the researchers’ instructions, and one lost to follow-up (M3). The final group composition comprised of 16 participants in the glucan group and 12 in the control group. **Figure 2** illustrates the flow diagram of participant selection and follow-up.

The glucan group exhibited an average age of 29.5 years, while the control group demonstrated an average age of 18.3 years. It is noteworthy that a discrepancy in the mean age of the groups was observed; however, this did not affect the outcomes, as age was not correlated with NAbs levels in the correlation analyses (Spearman and Pearson).

Thirty days after the prime immunization dose (M2), both groups exhibited the expected production of anti-S1 IgG, as stipulated by the immunization protocol. However, the levels of anti-S1 IgG differed between the two groups (*p* = 5.156^-10^), with the control group demonstrating higher levels (median - control: 2561.01; glucan: 1904.53). However, following the administration of the second dose (M3), a reversal of this profile was observed, characterized by a significant increase in anti-S1 production within the glucan group (*p* = 0.001237; median - control: 2611.18; glucan: 4040.98). With regard to anti-RBD IgG, which were also induced by the vaccine, the pattern was analogous, with elevated levels observed in the control group in M2 (*p* = 0.00000000476; median - control: 1192.06; glucan: 735.43). Conversely, in M3, the response was more pronounced in the glucan group (*p* = 0.002074; median - control: 877.95; glucan: 1452.75).

It is noteworthy that the ChAdOx1 immunizer does not result in the production of antibodies directed toward the viral nucleocapsid (N). However, in the serological evaluation of individuals not exposed to SARS-CoV-2 (as confirmed by the absence of anti-S1 and -RBD IgG in M1), the presence of anti-N IgG was detected as early as time M1 (prevaccination in 11 subjects, Control 5; Glucan 6). The mean levels were 138.9 ng/mL in the control group and 89.51 ng/mL in the glucan group. Over the course of the vaccination, it was observed that the anti-N levels in the control group increased to 252.58 ng/mL. In the glucan group, these levels declined (43.93 ng/mL).

In relation to the levels of NAbs produced (%IH), the paired analysis from M2 to M3 (a follow-up of different time points within the same group) demonstrated a substantial enhancement in functional antibodies in both cohorts, as anticipated, thus corroborating the pivotal function of the second dose in the generation of these functional antibodies, which have the potential to hinder the binding of the virus to the human ACE2 protein (Glucan Group: *p* = 0.00006104; Control Group: *p* = 0.009277).

**Figure 3:**
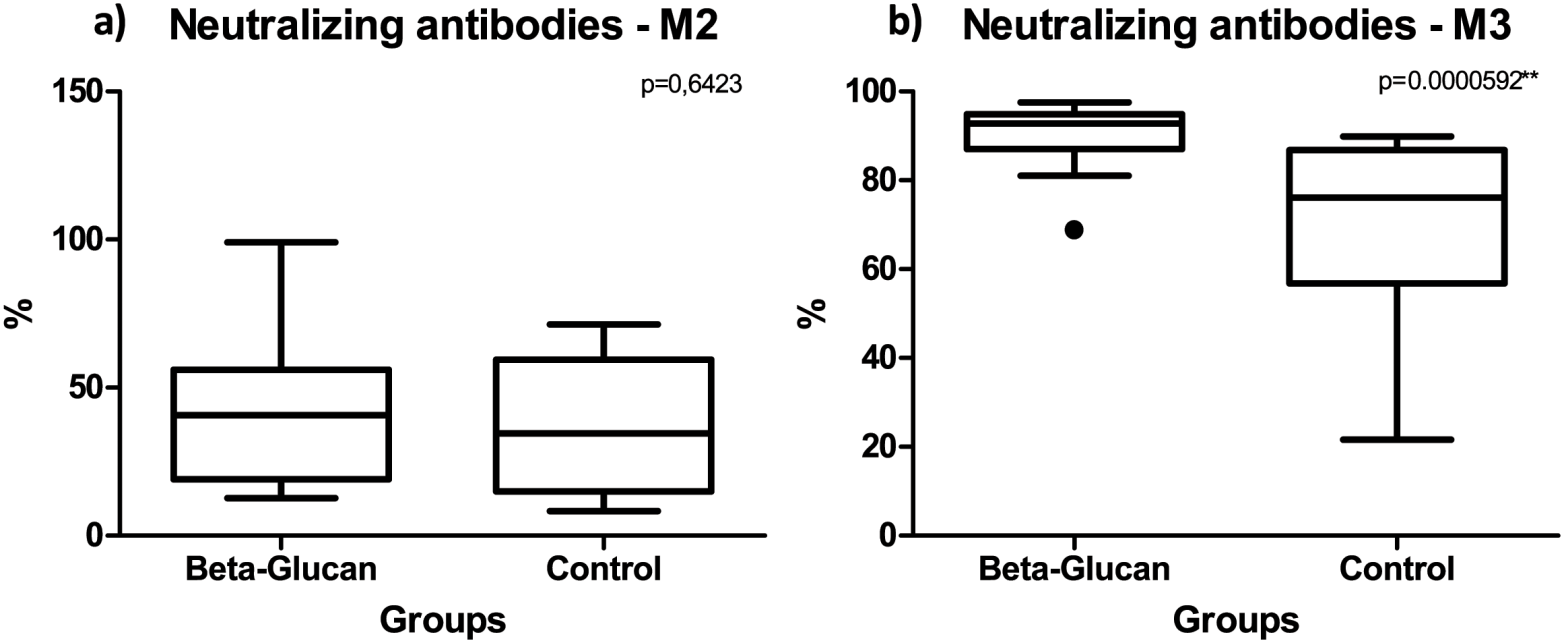
Comparison of neutralizing antibody (NAb) levels (%IH) in the control and glucan groups at two time points: **(A)** 30 days after the first vaccine dose (M2) and **(B)** 30 days after the second dose (M3). As can be seen, there was higher production of neutralizing antibodies in the glucan group than in the control group at both M2 and M3, but the increase was more significant at M3.

After one dose of the vaccine (M2) both groups produced NAbs. The values are represented as the median [P25-P75], respectively. The glucan group displayed a median value of 40.64% [21.96-53.14], while the control group exhibited a median value of 34.61% [18.89-51.65]. No discernible influence of supplementation was observed on the levels of these Nabs produced (*p* = 0.64). However, after the second dose (M3), the group glucan produced significantly higher levels of NAbs (*p* = 0.0000592), with a median of 92.81% [89.23-94.62], and the control group had 76.16% NAbs [59.58-82.46] (**Figure 3**).

**Figure 4:**
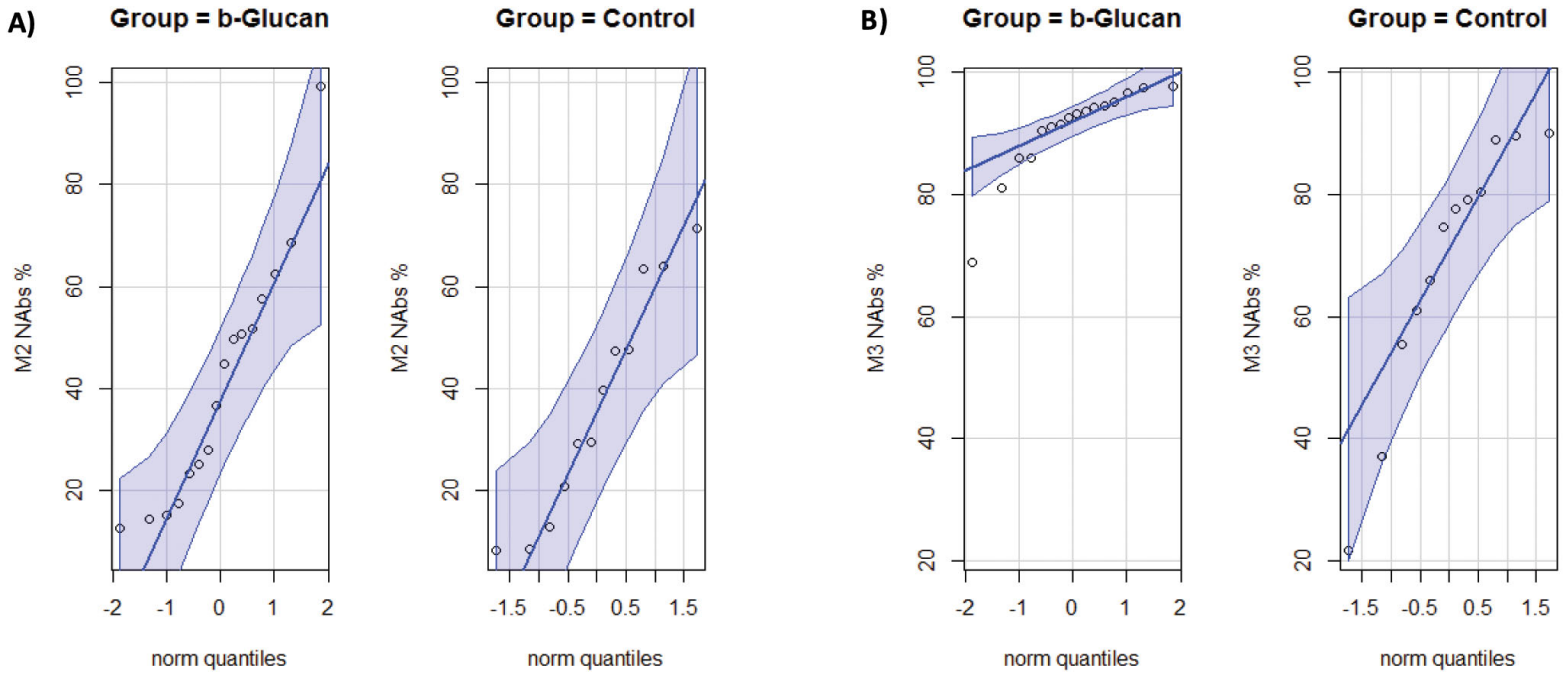
NAb levels (%IH) in the groups (median and IQR): **(A)** M2; **(B)** M3. A higher rate of NAb neutralization was observed in the glucan group in M3, which was characterized by homogeneity. A lower and more heterogeneous rate was noted in the control group.

**Figure 4** provides a different perspective on the NAbs levels obtained by the cohorts. In M2, production manifested heterogeneity and similarity across both groups (**Figure 4A**). It is noteworthy that, in M3, NAb production demonstrated elevated levels in the glucan group, accompanied by enhanced homogeneity, i.e., all supplemented individuals attained high neutralization rates with minimal variation between them. As demonstrated by the slopes of the lines in **Figure 4B**, which illustrate the median and interquartile range of NAb level (M3) in each group, this finding is substantiated. The dispersion proved to be narrower in the glucan group, as the participants exhibited elevated levels of NAbs. Conversely, the control group demonstrated a higher degree of heterogeneity in NAb production, resulting in lower levels of functional antibodies. In fact, certain participants within this group produced percentage of inhibition levels lower than 50%.

In the glucan group, individuals who produced higher levels of anti-S1 IgG after the first dose of the vaccine (M2) also produced higher levels of NAbs at M3 (*p* = 0.02236; Cor.: 0.57). This observation suggests that a directed immune response may occur even with the initial dose, leading to a more effective humoral response after the second vaccine dose.

The data revealed no correlation between the levels of anti-N IgG and NAbs in either group. However, a positive correlation was observed between the levels of anti-N IgG in M1 [measured pre-vaccination, i.e., possibly originating from common cold human coronaviruses (HCoVs)] and the production of anti-S1 IgG after second dose (*p* = 0.038; Cor.: 0.52), specifically in the glucan group.

B lymphocyte analysis demonstrates an increase in total B cells following the administration of the first vaccine dose (M2). This outcome was particularly pronounced in the control group (*p* = 0.0009766). Following the administration of the second dose (M3), there was a notable increase in the percentages of plasmablasts (*p* = 0.01669) and memory B cells (*p* = 0.03734) in the control group. A notable observation was the negative correlation between NAbs and plasmablast levels post-second dose, exclusively in the glucan group.

## 4 Discussion

The influence of sex on immune responses is a critical factor in understanding disparities in infectious disease outcomes, vaccine efficacy, and autoimmune prevalence. These disparities can be ascribed to a combination of hormonal, genetic, and environmental factors (48, 49). Therefore, in an effort to mitigate potential biases, the population of this study was restricted to male participants, thereby reducing the need for a more substantial study population size.

The ChAdOx1-S/nCoV-19 vaccine uses a replication-deficient adenoviral vector that expresses the SARS-CoV-2 spike protein gene, prompting the immunological response and production of memory cells. The Spike protein (S) is necessary for the virus to enter host cells because it contains the receptor-binding domain (RBD), which interacts with angiotensin-converting enzyme 2 (ACE2) on the surface of host cells. Anti-S/anti-RBD neutralizing antibodies block viral fusion mediated by the S protein (RBD portion) of SARS-CoV-2, preventing its interaction with ACE2 of the host cell (16, 50, 51). The present study demonstrated that both groups exhibited efficient production of anti-S and anti-RBD IgG following immunization (M2 and M3), in accordance with the expected outcomes (52). However, the levels of these antibodies differed between the groups. The control group demonstrated higher levels after the first dose, while the glucan group exhibited increased levels after the second (7, 49).

Individuals with anti-N (M1) IgG exhibited diminished levels of these immunoglobulins during immunization when b-glucan was utilized as a supplement. This finding is particularly noteworthy in light of the fact that the ChAdOx1-S/nCoV-19 vaccine does not utilize the nucleocapsid (N) protein as an antigen. Conversely, subjects in the control group exhibited elevated anti-N levels during the post-vaccination follow-up period. As nucleocapsid is a conserved protein among different species of coronaviruses and is recognized by partially cross-reactive HCoVs antibodies, it can be hypothesized that these antibodies originate from other HCoVs that also lead to seasonal respiratory tract infections. The observations reported here suggest the potential for a more targeted immune response to the vaccine in the glucan group. This response may include the inhibition of memory antibodies from other HCoVs and the enhancement of the expansion of antibodies directed against specific vaccine antigens, here, namely, Spike and RBD (53).

β-glucan supplementation has been shown to induce TRIM, enhancing adaptive responses without directly suppressing antibody production against other antigens. Netea et al. (31) demonstrated that this innate memory is not antigen-specific but can polarize immune responses toward Th1/Th17 profiles, thereby influencing antibody subclasses. Similarly, Quintin et al. (18) noted that nonspecific innate activation may compete for immunological resources, modulating the intensity of humoral responses to multiple antigens. More recently, it was reported that *Saccharomyces cerevisiae* β-glucans strongly promote trained immunity and Th1/Th17 polarization, accelerating antibody production (54). Collectively, these findings indicate that β-glucans modulate humoral responses through resource competition and immune polarization rather than direct suppression.

Both groups had their highest levels of NAbs after the second vaccine dose (M3) compared with the first dose (M2). However, the glucan group exhibited higher production (median = 92.81%), indicating that immunomodulation induced a more robust response capable of producing higher levels of neutralizing antibodies in response to the vaccine stimulus. Along with the increased production of NAbs, we observed a more uniform humoral immune reaction in the glucan group (displayed in **Figure 4B**), while in the control group NAb production was more varied, with wider differences between individuals and a lower minimum level of functional antibodies. This observation of variable NAb levels has also been reported in individuals convalescing from SARS-CoV-2 infection (49, 55).

The NAb rate recorded in the control group following the administration of the second dose of the ChAdOx1 vaccine (median = 76.16%) demonstrated comparable results to those documented in studies assessing the neutralizing capacity of different immunizers against SARS-CoV-2 over time, with a median of 76.7% (15 days) and 81.6% (30 days), respectively (8, 49, 55–57). These results are consistent with previous research outcomes, indicating that our study population is representative of the general population.

The findings indicated a positive correlation between anti-N IgG (M1) levels and anti-S1 production in M3. However, these antibodies were not correlated with neutralizing antibodies. This observation indicates that prior exposure to other HCoVs does not influence the magnitude or direction of the humoral immune response, as evidenced in the glucan group. Consequently, it can be inferred that glucan supplementation was more efficacious than cross-immunity from other HCoVs (53). Supplementation with β-glucan induced an acceleration of the immune response to the stimulus (vaccine), making it more efficient at developing functional antibodies. The ability of β-glucan to induce TRIM, directly stimulating and amplifying immune responses upon secondary stimulation, has been described in several studies (19, 25, 36, 58). Individuals in the glucan group exhibited a NAb level (median = 92.82%) analogous to that of individuals who had previously recovered from SARS-CoV-2 and had received complete immunization (19, 30, 55, 59).

A parallel study performed by our research group (53) evaluated individuals who had received two doses of ChAdOx1 without prior exposure to SARS-CoV-2 (designated as the “naïve+vax” group) or with infection prior to vaccination (referred to as the “COVID+vax” group). The findings revealed that NAbs levels (median) were observed to be 81% (P25 = 0; P75 = 96) and 98% (P25 = 90; P75 = 99), respectively, in these groups. The response profile observed in individuals who had previously contracted SARS-CoV-2 and subsequently received full immunization exhibited notable similarities to that obtained in the group supplemented with β-glucan, resulting in a more homogeneous humoral response, as evidenced by the elevated levels of NAbs. These outcomes are further substantiated by the assessment of neutralizing antibodies in unvaccinated convalescent individuals (immunity exclusive to the disease), which was also measured in this additional study. Levels of NAbs were found to be significantly lower and more heterogeneous (median 38%; P25 = 5; P75 = 70), indicating that the β-glucan supplementation generates higher levels of NAbs than the disease itself. It is noteworthy that 26.6% of the naïve+vax group exhibited an absence of response to the vaccine stimuli or a partial response, a result that aligns with the observation reported by Eliçabe et al. (49). This observation indicates the potential for prior supplementation with β-glucans to impact the humoral response, thereby reducing the incidence of response failures and the subsequent necessity for vaccine boosters (32, 49, 59).

Unlike the glucan group, the control group exhibited elevated levels of B lymphocytes at M2. This increase may signal clonal expansion. Additionally, no correlation was observed between total B lymphocytes and neutralizing antibody levels. It has been posited that glucan may have exerted its effects through qualitative rather than quantitative optimization of the humoral immune response. This would induce differentiation of B lymphocytes into antibody-producing cells at an early stage, even in the absence of a significant numerical increase. These data corroborate our finding of specific immunoglobulin production against the virus, especially the production of neutralizing antibodies, which were higher in the glucan group. As Ikewaki et al. have demonstrated, β-glucans have been shown to induce B lymphocytes to differentiate and produce antibodies (36).

A study evaluating leukocyte subpopulations in peripheral blood observed that the increase in plasmablasts after vaccination with the ChAdOx1 vaccine was accompanied by a late increase in memory B cells after the first dose (60). We hypothesize that this process is accelerated by TRIM with β-glucan, allowing the increase in plasmablasts and generation of memory cells to occur earlier in supplemented individuals. This hypothesis is supported by our analysis of subpopulations of B lymphocytes at M3, which showed that the percentage of plasmablasts was significantly higher in the control group than in the glucan group. We observed a negative correlation between plasmablasts and NAbs in the glucan group (Cor: -0.77), i.e., the fewer the circulating plasmablasts, the more the antibodies with neutralizing capacity.

During the ontogeny of plasma cells, memory B cells that have matured through primary immunization can differentiate into plasmablasts or plasma cells when stimulated a second time (secondary immunization). These cell subpopulations have different roles in antibody production. Plasmablasts are responsible for the initial production of antibodies, while plasma cells produce mature antibodies with a stronger binding affinity between antigen and antibody (61, 62). The data indicate that the glucan group had already entered this maturation stage (possibly at M2), on account of its high levels of circulating NAbs. In the control group, this production could occur later, while the cells are still differentiating into plasmablasts. This would result in a subsequent production of high-affinity antibodies. These results corroborate studies that show evidence that dendritic cells stimulated with β-glucans are more efficient at supporting the responses of B cells and in the production of NAbs, which would help to transition the early innate immune response toward a long-term adaptive response of β-glucans (25, 63).

The TRIM phenomenon induced by β-glucan, even in the face of infection, may be similar to that provided by BCG (19, 26, 58, 64, 65), a vaccine that generates robust immune stimuli and is one of the most studied models of TRIM. In response to BCG, CD4+ and CD8+ memory cells can be activated in an antigen-nonspecific manner, increasing the Th1 and Th17 responses, in addition to inducing higher antibody titers and T-cell responses, influencing the responses generated by vaccines administered later against hepatitis, poliomyelitis, and pneumococcal conjugate vaccines (64), which justifies the early administration of BCG in newborns. Although it seems to act similarly to the BCG vaccine, the TRIM induced by β-glucan seems to be more relevant in the primary vaccination course, inducing greater production of specific antibodies with the first dose. These data reinforce the hypothesis raised in our previous study of the hepatitis B vaccine, in which glucan supplementation was performed before the booster dose (fourth dose) and did not induce a significant difference in the production of antibodies when compared to placebo, possibly due to the immunological memory already acquired by earlier vaccinations. Based on these observations, we set out to evaluate β-glucan influence on primovaccination (66).

In the context of the COVID-19 and the need to update existing vaccines due to new viral variants, supplementing with β-glucan at each revaccination may be beneficial. In addition, it is believed that partial vaccination and/or incomplete immunization may lead to the development of subneutralizing antibody titers, which may favor the emergence of escape variants, affect the efficacy of the vaccine (67), or even act as a mechanism that facilitates infection, as observed in the antibody-dependent enhancement phenomenon, which has been well documented in other viral infections, such as dengue (30, 68). This problem can be minimized with oral β-glucan supplementation, which induces an acceleration of the immune response as well as higher functional antibody titers.

A recent study presented experimental results of a new vaccine that uses a chimeric protein that includes the RBD and N antigens of SARS-CoV-2, indicating that it can stimulate the generation of effector and memory T cells against SARS-CoV-2, but not the production of antibodies. The authors suggest that this new vaccine can be used in combination with other existing vaccines, which induce the production of antibodies, potentiating the generated immune response (15) and being an alternative for protection against other variants (11, 13, 69). In this context, the concomitant use of β-glucan could contribute to this vaccine association, providing a more orderly, homogeneous, and robust immune response. Tsang et al. proposed altering baseline immunity through the administration of immunomodulators prior to vaccination to improve efficiency and optimize vaccine performance (12). Moreover, the potential of TRIM as an isolated strategy to improve the baseline status of immunity has been considered, with the spread of emerging pathogens in the early stages of new epidemics being reduced in order to prevent disease exacerbations and mortality in high-risk groups until specific treatments and/or immunizations are developed (12, 70).

Understanding innate immune memory is fundamental, as the cellular and molecular mechanisms of TRIM can contribute to strategies for developing new generations of therapies and vaccines that combine the induction of TRIM with adaptive immune memory, with the aim of achieving a broad spectrum of pathogen protection for different variants, better rates of effectiveness and durability (12, 15, 19, 20, 36, 69, 70).

Study Limitations: The reduced sample size is attributable to two independent contextual factors: (i) the nationwide scarcity of SARS-CoV-2–specific supplies during the study period, with an average importation time exceeding six weeks, which limited the immediate availability of essential materials; and (ii) the restricted recruitment window, as the municipality was included in the effectiveness study of the ChAdOx n-CoV vaccine, with mass immunization scheduled to begin on May 16, 2021, thereby rapidly reducing the pool of eligible unvaccinated participants. In light of these constraints, conducting the study with a smaller sample was considered both methodologically appropriate and ethically justified. This approach enabled the generation of initial parameters (effect size, variability) to inform future large-scale clinical trials without compromising participant safety or the exploratory validity of the results. Moreover, the longitudinal nature of the sample collection in our study design is advantageous, as it enables the utilization of each participant as their own control, both before and after the administration of the vaccine. Considering the well-established influence of sexual activity on immune response, it is not feasible to extrapolate the findings of this study to women. Another limitation pertains to the inclusion of different age groups, as the study did not encompass children or the elderly, two populations in which the assessment of the applicability of β-glucans in the context of post-immunization immunity would be particularly pertinent. Despite the evidence from the present study that suggests the indirect suppression of preexisting anti-N by supplementation with β-glucans, as reported in the literature, it remains inconclusive due to the absence of an assessment of the molecular mechanisms involved in this effect.

## 5 Conclusion

In summary, the combination of oral β-glucans and the ChAdOx1-S/nCoV-19 vaccine led to effective immunomodulation, which resulted in a more robust and specific humoral immune response. β-glucan-induced TRIM is a relevant public health strategy since it can guide innovations in immunization protocols, influence the number of doses, and have an economic impact. It can also contribute to the efficacy and durability of the vaccine response, benefit poor responders, the elderly, and immunodeficient individuals, and favor cross-protection against viral variants. Further research is needed to investigate β-glucan as a vaccine-adjuvant agent. These studies should examine its impact on immunological responses to vaccines across different age groups and in immunocompromised populations, as well as its potential to promote long-term immunity and cross-protection against other viral variants.

## Data Availability

The raw data supporting the conclusions of this article will be made available by the authors, without undue reservation.
